# Laser fabrication and evaluation of holographic intrinsic physical unclonable functions

**DOI:** 10.1038/s41598-022-06407-0

**Published:** 2022-02-21

**Authors:** Aggeliki Anastasiou, Evangelia I. Zacharaki, Anastasios Tsakas, Konstantinos Moustakas, Dimitris Alexandropoulos

**Affiliations:** 1 Department of Materials Science, University of Patras, 26504 Patras, Greece; 2 Department of Electrical and Computer Engineering, University of Patras, 26504 Patras, Greece

**Keywords:** Optics and photonics, Applied optics

## Abstract

Optical Physical Unclonable Functions (PUFs) are well established as the most powerful anticounterfeiting tool. Despite the merits of optical PUFs, widespread use is hindered by existing implementations that are complicated and expensive. On top, the overwhelming majority of optical PUFs refer to extrinsic implementations. Here we overcome these limitations to demonstrate for the first time strong intrinsic optical PUFs with exceptional security characteristics. In doing so, we use Computer-Generated Holograms (CGHs) as optical, intrinsic, and image-based PUFs. The required randomness is offered by the non-deterministic fabrication process achieved with industrial friendly, nanosecond pulsed fiber lasers. Adding to simplicity and low cost, the digital fingerprint is derived by a setup which is designed to be adjustable in a production line. In addition, we propose a novel signature encoding and authentication mechanism that exploits manifold learning techniques to efficiently differentiate data reconstruction-related variation from counterfeit attacks. The proposed method is applied experimentally on silver plates. The robustness of the fabricated intrinsic optical PUFs is evaluated over time. The results have shown exceptional values for robustness and a probability of cloning up to $10^{-14}$, which exceeds the standard acceptance rate in security applications.

## Introduction

Security of information and goods is a priority in everyday practice, that is protected by a strict legislative framework and various technological solutions to prevent bridge and unlawful access. Counterfeiting is the most common bridge of security that impacts economy and public health. For example, counterfeiting pharmaceutical products can be lethal^1^. Similarly, counterfeiting parts in a production line usually leads to under-performance or even failure of the end product^2–5^. Among the various security primitives, researched and developed, Physical Unclonable Functions (PUFs)^6,7^ have the potential to become the golden security standard^8^. A PUF is a unique physical entity almost impossible to duplicate or clone. The uniqueness of its structure stems from the random physical phenomena introduced during manufacturing^9^. PUFs can be broadly, categorized as *strong* and *weak* PUFs depending on how easily they can be compromised and *intrinsic* and *extrinsic* depending on whether the PUF entity is an integral part of the good (intrinsic)^10^ or not (extrinsic)^11^. Any random physical process is a potential candidate for the realization of PUFs. Indeed, there is already a plethora of research accounts^12–19^ that include exotic solutions like aerogels^20^, Raman tags^21^, plasmonic nanopapers^22^ wrinkles on glasses^23^, perovskite fluorescent dots^24^ or even edible unclonable functions^25^ to name a few. The PUF scenery in the literature is evolving fast necessitating taxonomy activities like the one presented by McGrath and co authors^12^. In Ref.^12^ the various PUF concepts are catalogued in terms of nature and physical process. Along similar lines, Gao et al.^26^ have recently reviewed PUF technologies from the viewpoint of applications for PUFs considering unreliability and security issues.

The overwhelming number of PUF realizations reflects the plurality of physical processes that can be exploited. In most PUF demonstrations, although security of goods and services is evoked, it is surprising that these are rarely assessed in terms of practical implementation both in terms of industrial compatibility for upscaled production and PUF evaluation apparatus for Challenge Response Pair (CRP) behavior at the end user. In this context, it is hard to envisage how the various demonstrations like^20,23,24^ mentioned above will be incorporated in an industrial fabrication of everyday goods or how bulky machinery, such as the spectroscopic equipment required in Ref.^21^, can be used for read out of PUF response somewhere in the supply chain. Despite the interesting physics and science entailed in the various PUF demonstrations, it will be the tradeoff between built-in complexity, hence unclonability, and industrial scalability, hence cost effectiveness, that will determine the viability of each PUF scheme that can be further specified for the targeted application.

In this sense, it is instructive to revisit PUFs requirements, namely uniqueness, robustness and unclonability^9^. Given the practical importance of PUFs one should elaborate this list with the requirements of practical implementation and cost effectiveness. These translate to compatibility with existing industrial fabrication processes and long-term stability of the PUF responses. Part of these requirements may be relaxed or emphasized depending on the application. For example, the so called weak PUFs^12^, are easy to fabricate yet they are vulnerable to machine learning (ML) attacks (see Table 1 of^26^), hence the name. Similarly, extrinsic PUFs^12^ are easier to fabricate, however they are by default more vulnerable compared to intrinsic PUFs since e.g. an attacker may interfere and replace them. A legitimate criticism concerns the fabrication methods used for the generation of PUFs, especially of strong PUFs, since in the vast majority these are not compatible with the fabrication methods used for the commodity goods. Therefore, it does not come as a surprise that electronic intrinsic PUFs are gaining ground compared to other PUF implementations despite the fact that they are less secure, since they can be fabricated with the well-established CMOS technology. Aside from fabrication, another challenge for the PUF technology is that the PUF entity needs to be an integral part of the item that it aims to protect. This is still only partially met: in most present-day implementations the PUF is an add-on rather than part of the item and this compromises PUFs robustness. In silicon PUFs (e.g. SRAM PUFs^27^ or MEMS PUFs^28^), the PUF is indeed part of the item, yet these are weak PUFs. Electronic PUFs can also be considered as part of the item, yet they are vulnerable to machine learning attacks^29^.

Interpreting these trends, one concludes that in present day PUF scenery, fabrication simplicity is favored over security, and extrinsic PUFs over intrinsic PUF. The next natural technological step is the development of intrinsic strong PUFs that are simple to fabricate and to evaluate. Despite the intensive research activity^30^ a *low-cost* *intrinsic strong PUF* technology that is industrial friendly is still elusive, hindering large-scale deployment. In this paper, we demonstrate for the first time to our knowledge strong intrinsic Computer-Generated Holograms (CGH)^31^ as PUFs (CGH-PUFs) on metallic goods that are fabricated using laser-based techniques, compatible with industrial fabrication processes. We exploit the randomness of the laser matter interaction to demonstrate unique optical response profiles of *intrinsic* PUFs that are robust, unclonable, reliable and scalable. In doing so, we use an industrial nanosecond IR fiber laser for the fabrication of holograms on silver surfaces and a simple evaluation setup to record the reconstruction upon illumination with a standard laser source. This fast, facile, and low-cost laser engraving fabrication process of CGH-PUFs outperforms more complex and time-assuming processes of other PUF fabrication methods. It is the less precise control of the laser matter interaction induced by the IR nanosecond laser that delivers the necessary randomness for the PUF’s purposes in the micro/nanostructures on the samples. Moreover, we develop an advanced novel Machine Learning (ML)-based evaluation algorithm for the accurate PUF authentication, that adds up to robustness. The image normalization and registration processes as well as the computation of sophisticated similarity metrics lead to high authentication performance: the PUF evaluation across different days reveals enhanced robustness and a low probability of cloning.

## Optical PUFs design: system architecture

### Laser fabrication of holographic PUFs

Unlike previous approaches in the literature, here we revisit the use of laser-based techniques for the fabrication of PUFs. The necessary randomness is provided by the highly complicated nature of laser matter interaction^32^. There are several advantages in using laser-based techniques: lasers are well adopted in fabrication processes for industrial production^33^, hence PUF fabrication can be readily upscaled. The use of lasers allows for the fabrication of intrinsic PUFs in a one-step process, so that the good to be authenticated is the PUF itself. We benefit from the uncontrollable nature of some of the phenomena associated with processing of materials with nanosecond laser pulses to engrave PUFs in the form of holograms. Especially for nanosecond lasers, the thermal process dominates the laser pulse material interaction with pronounced extended Heat Affected Zone (HAZ). Laser ablation is accompanied with *uncontrollable* melting and solidification, redeposition and shockwaves as well as thermally induced defects^34–36^.

**Figure 1:**
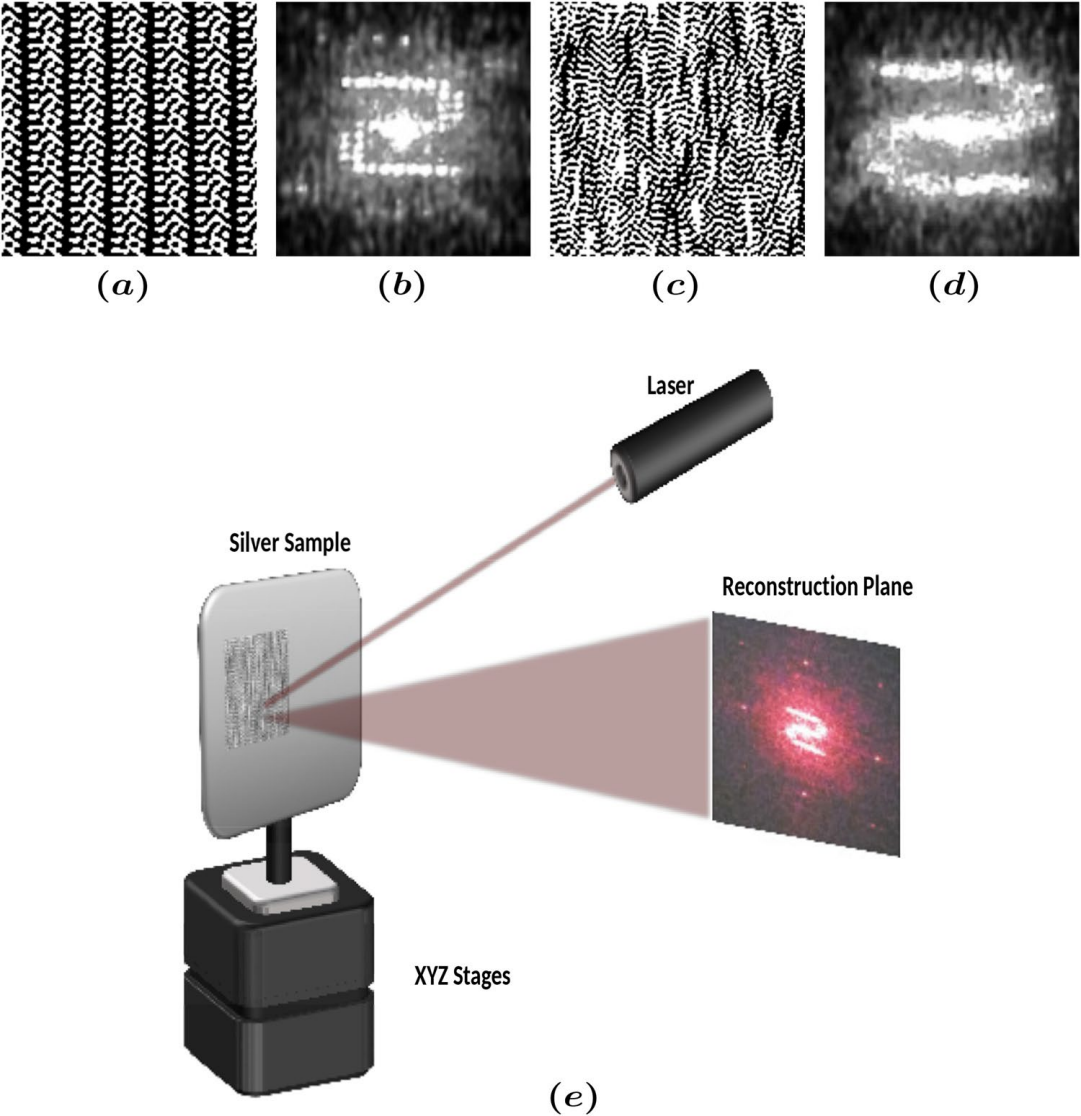
Holographic masks (**a**,**c**) and reconstructed images (**b**,**d**) for beam *Splitter* (**a**) and beam *Diffuser* (**c**) structure design of a CGH and the reconstruction setup (**e**).

In essence, our method of hologram engravings of PUF is a speckle-based technique. Speckle-based Optical PUFs^37^ are strong PUFs, i.e. unclonable and immune to attacks, that rely on the generated speckle from a random structure upon illumination. Speckle^38^ is a form of coherent noise that results from destructive and constructive interference of scattered light from microstructures on the laser processed surface. It is emphasized that our technique differs distinctively from previously reported techniques^39^ that use the engravings of holograms as low security tags. In our approach, the PUF is generated by the random information from the disorder produced upon laser processing with the nanosecond pulsed laser while the meaningful information carried by the hologram acts as anchor points that relax the sensitivity to probing thus improving dramatically robustness. Therefore, our technique overcomes the severe limitations of present-day speckle-based optical PUF approaches that they are “notoriously sensitive to probing and environmental variations”^40^. We choose to model CGH both in beam *Diffuser (BD)* design and beam *Splitter (BS)*^31^ (Fig. 1). The *BD* design is more demanding in terms of pixel size and therefore is less tolerant to potential variations during the fabrication process, hence most suitable for optical PUFs.

For the validation of the method, we have fabricated twenty (20) CGH on the same silver plate with the exact same conditions, namely pulse duration, power and passes, emulating an industrial production line in ambient atmosphere. Half of them (10) were engraved with a beam *Splitter* holographic mask and the rest with a beam *Diffuser* holographic mask. No special room conditions were applied (e.g. regulated temperature or air filtering). Analysis of these engravings reveals similarity in the low spatial frequency content, whereas the high frequency content differs significantly. It is the latter that ensures the requested unclonability. It is interesting to note that there are differences within the same CGH engraving: apart from the randomness during the laser ablation process, the heating of the sample during laser processing alters the reflectivity and therefore the conditions for ablation. This effect was highlighted in Ref.^41^ where a machine learning methodology was proposed for the minimization of the consequences of sample thermalization on the CGH quality. Obviously, here we follow the opposite route.

The proposed method offers many degrees of freedom for additional randomness and unpredictability in the fabrication process that are beneficial for PUFs. Sources of randomness are (a) instabilities in the intensity of the laser source itself, (b) the unstable polarization of the laser sources, (c) uncertainties in the galvo mirrors when operating at low frequencies. Furthermore, often dyes are used to enhance the absorption and suppress reflection upon laser irradiation. The manual application of dye introduces some experimental variation leading to increased uncertainty.

**Figure 2:**
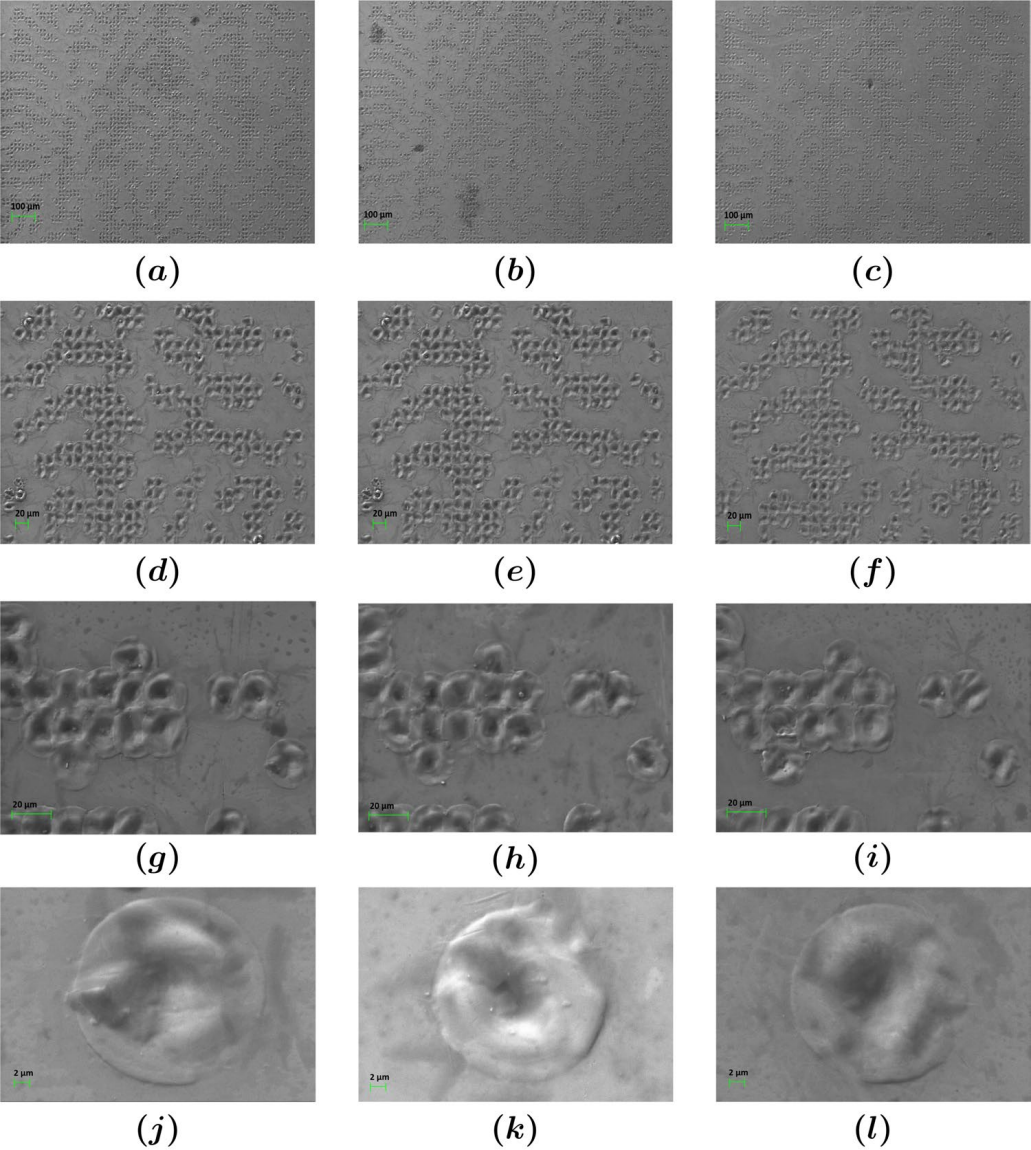
SEM images for three indicative engravings of beam *Diffuser* structure design (BD3, BD1 and BD5 respectively). Each column corresponds to different engraving and each row to different magnification: (**a**–**c**) 220X, (**d**–**f**) 600X, (**g**–**i**) 1.84KX and (**j**–**l**) 7.45KX.

For the image reconstruction, we used the experimental setup of Fig. 1e. The reconstructed image contains both the meaningful information, in this case the letter “S”, as well as the speckle noise. Irregular and uncontrollable nano/microstructures result upon laser ablation with nanosecond pulsed lasers as discussed above. The higher the irregularity of the surface, the more pronounced is the speckle noise. Figure 2 shows scanning electron microscopy (SEM) images of the fabricated CGHs, captured with Zeiss EVO MA 10. The desired speckle is generated by the nano/microstructures in and around the crater. An example of a target image with the corresponding phase mask and obtained reconstructed image are shown in Fig. 1.

## Authentication methodology

**Figure 3:**
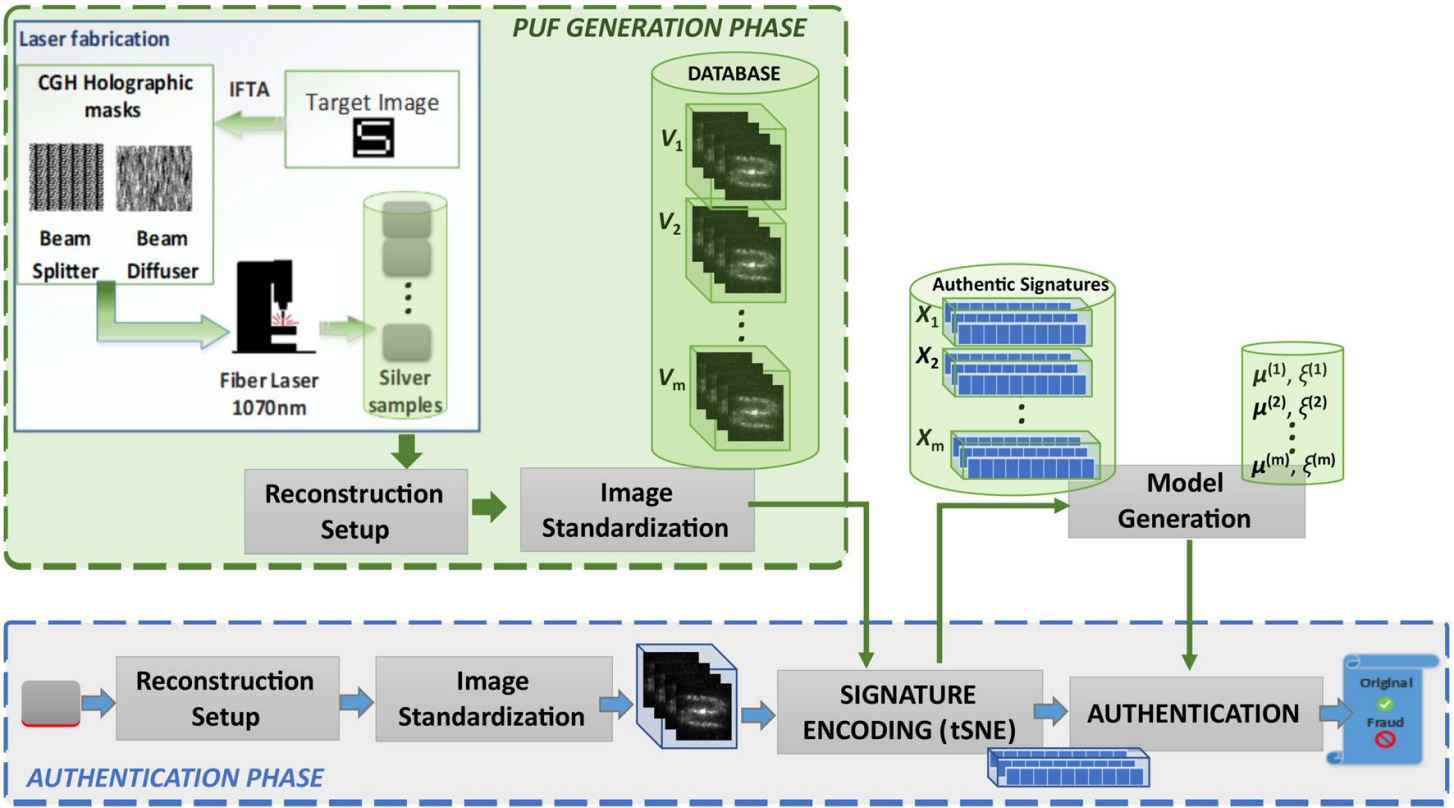
Schematic diagram of our methodology including laser fabrication, signature encoding and authentication phases.

As described above, the response of our laser fabrication and image reconstruction system consists of a holographic image with a noisy, but structured, central shape (representing a selected letter) merged with a speckled background. Every new illumination of the engraved structure produces a unique image due to the random light scattering effects. The authentication challenge thereby can be cast as a method that is able to identify engraving-related image effects irrespectively of the image reconstruction variation and random noise. This method should be powerful enough to distinguish images obtained from the most similar engravings, e.g. as the ones produced by the same holographic mask of a CGH, under the most challenging conditions, i.e. in loosely controlled settings. To address these challenges, we follow a methodology that first minimizes the acquisition-related data variation (misalignment of images) and then uses semi-supervised learning techniques to model the variation of the reconstructed images and encode each of them through a unique descriptor in a low-dimensional space, where relative distances can be computed. By using this descriptor in a metric space, we can assess the engraving’s authenticity, quantified as image similarity versus the original images acquired at baseline. The steps of our authentication methodology are illustrated schematically in Fig. 3.

In more details, the PUF generation phase of the proposed method includes the fabrication of the engraving, the holographic image acquisition and the spatial standardization of the reconstructed images. This latter procedure involves linear image registration, cropping and conversion to grayscale. In the signature encoding phase, we encoded the images with fewer variables using a non-linear dimensionality reduction technique. A set of multiple PUFs is used for the extraction of authentic signatures and stored to a database to be used at the authentication phase. To evaluate the authenticity of a CGH-PUF, we repeat the steps of image acquisition, standardization and dimensionality reduction, where in the latter, the new responses are combined with the responses from the database in order to extract the signatures of the testing sample and compare it against the existing (authentic) ones. If the *authentication score* exceeds a threshold, the testing sample is considered as genuine, otherwise as counterfeit.

### Image standardization

As a first step, the colored (RGB) images obtained with the webcam are transformed to grayscale and quantized as 8-bit unsigned integers in the range of 0 to 255, to reduce memory requirements and processing time. To evaluate authenticity and robustness in the creation of PUFs the definition of a metric is required that allows to quantify similarity between a test and a reference image sample. A point-wise comparison would not reveal similarities even for images obtained from the same engraving because of misplacement during image acquisition. To remove the effect of misplacement during image acquisition we spatially normalize each image to a common (reference) space. For this purpose, we exploit the fact that our images contain a distinct pattern (the holographic shape) that can guide an image registration algorithm in the mapping process. In detail, let $\Omega _S$ and $\Omega _R$ denote the domain of a source and a reference (template) image, respectively, and $I_S(\mathbf{p} )$ the intensity of the source image at pixel $\mathbf{p} \in \Omega _S$ and $I_R(\mathbf{q} )$ the intensity of the reference image in pixel $\mathbf{q} \in \Omega _R$. Image registration is a problem of calculating a spatial transformation $T:\Omega _S \rightarrow \Omega _R$ that brings the two images into alignment, such that the same structures spatially coincide. This is accomplished by maximizing a similarity criterion that quantifies the level of alignment between the images. We used as geometric mapping function an affine transformation that accounts for translation, rotation, scaling, and shearing effects in order to ensure that the reconstructed images which were used as input to our method, would not be affected from any possible instabilities on the reconstruction setup. For a two-dimensional spatial domain, the affine transformation is expressed with 7 parameters (1 for rotation, 2 for translation, 2 for scale and 2 for shear). Since the image to be mapped to the reference space is of the same modality with the template (i.e. a separate template is used for images with beam *Diffuser* or beam *Splitter*), we selected as similarity criterion the mean square error, which provides an average pixel-wise difference.

Subsequently, the images are centrally cropped to keep the most distinctive part of the reconstructed images and exclude the surrounding noise, while reducing the image dimensionality. Each two-dimensional image is then vectorized and stored as a one-dimensional vector, denoted with $\pmb {v} \in {\mathbb {R}}^D$, where *D* is the number of pixels after cropping. No additional filtering is performed on the images because we have no intention of eliminating the background noise around the central structured pattern (symbol ‘S’ in our case). In fact, the uniqueness of this speckle noise is the basis for the authentication process, since it acts as the signature of the engraving, as presented in corresponding literature^37,42^.

### Signature encoding with t-SNE

Any authentication methodology is based on evaluating similarity or distance between a new sample and the reference (authentic) data. However, in our case the number of variables (pixels per image) is very large, and in such cases, Euclidean distances between different pairs of samples become numerically similar and lose their usefulness (*curse of dimensionality* problem). This problem is addressed with the use of linear or non-linear dimensionality reduction (DR) techniques that allow to extract a small number of principal features and thus represent the data in a lower dimensional space. We examined several DR techniques^43^ and selected the T-distributed Stochastic Neighbor Embedding (t-SNE)^44,45^ that exhibits several advantages, as will be explained next. T-SNE is a variation of the Stochastic Neighbor Embedding (SNE) and, as a manifold-learning technique, it examines the inherent local structure in an unsupervised way in order to map them in a low-dimensional Euclidean space where data comparison is feasible. Its main advantages over other DR techniques are that it reveals data that lie in multiple (different) clusters or manifolds and also it reduces the tendency to crowd points together at the center. While other techniques are appropriate for single continuous manifolds, t-SNE can extract clustered local groups of samples, thereby allowing to disentangle a dataset that comprises several manifolds at once. This is beneficial for the current problem as we expect that images acquired from different engravings will belong to different manifolds.

Assuming that $V = \left\{ \pmb {v}^{(i)}_j\right\}$, $V \in {\mathbb {R}}^{m \times D}$, is the set of *m* registered and vectorized images acquired from a small number of engravings (indexed by the superscript (*i*)) using multiple reconstructions for each engraving (indexed by the subscript *j*). In order to ensure equal representation of each engraving, we assume that approximately the same number of reconstructions is available from each engraving. We apply t-SNE to extract for every reconstructed image *j* of engraving *i* a lower dimensional feature vector $\pmb {x}^{(i)}_j\in {\mathbb {R}}^{d}$ (with $d<<D$), and use those feature values as the CGHs’ signature. We used maximum likelihood estimation (MLE) to estimate the dimensionality *d* of the embedding space, which led to the selection of $d=10$ features for signature encoding. The obtained set of signatures of all processed CGHs-PUFs, $X = \left\{ \pmb {x}^{(i)}_j\right\} , X \in {\mathbb {R}}^{m \times d}$, is stored and used later for verification of authenticity of a new test sample.

Moreover, we may clarify that t-SNE, as all manifold embedding techniques in this category, cannot be applied for signature extraction of a new test sample using only the data of this new sample. The new data are concatenated with the existing database in order to compute joint probabilities. This is an inherent limitation of non-linear DR techniques that causes an increase of computational cost during the inference (authentication) phase.

### Authentication rule

In this study, we formulated the authentication task as a one-class classification (OCC) problem^46,47^, in which information is provided only for one group of observations (target class) and this target class needs to be distinguished from all other possible classes, considered as non-targets. We followed an OCC approach (instead of two-class problem) for two reasons. First, this method suits perfectly the PUF’s encoding problems due to the difficulty of collecting attack or intrusion data from all possible attack scenarios against an application. The second reason relates to the algorithm selected for the extraction of the data signatures, i.e. t-SNE. Let us note that, while t-SNE retains useful local structure—such that data points that cluster together in the final embedding most probably were also very similar in the original high dimensional space (which are expected to be data from the same engraving)—it does not explicitly preserve global structure. Therefore, no inference can be drawn about data similarity in the original space for data points that appear far away from each other in the final embedding. Based on this OCC formulation, a different classification model (authentication rule) will be fitted to the data signatures of each engraving.

There are various approaches of OCC^47,48^ which use, for instance, a Gaussian model or a mixture of Gaussians models^49^, Support Vector Data Description (SVDD)^50^, Parzen or Naive Parzen density estimators to estimate and threshold the density of the target data^51^. In our approach, we capture the acquisition-related data variation of each authentic engraving by acquiring a number of different image reconstructions with varying conditions. If $X^{(i)}=\left\{ \pmb {x}^{(i)}_1, \pmb {x}^{(i)}_2, \ldots ,\pmb {x}^{(i)}_n \right\}$ is the set of *n* feature vectors for engraving *i*, the aim is to calculate a decision function *f* that predicts whether a new image sample $\pmb {y}$ has been acquired from engraving *i* (i.e. is authentic) or is an outlier. The likelihood of a sample $\pmb {y}$ to be an outlier is assumed to be proportional to its distance from the observed dataset $X^{(i)}$. Thresholding of the likelihood converts the continuous distance function to a binary discriminant function, with a positive label indicating authenticity and a negative fraud, as expressed below:

1 $$\begin{aligned} f(\pmb {y}; X^{(i)}, \xi ^{(i)}) = {\left\{ \begin{array}{ll} 1\, (authentic), &{} \text {if } ( d(\pmb {y}, X^{(i)})\le \xi ^{(i)}\\ -1\, (fake), &{} \text {otherwise}, \end{array}\right. } \end{aligned}$$

where $d(\pmb {y}, X^{(i)})$ is a (sample-to-cluster) distance metric and $\xi ^{(i)}$ a selected threshold. This simple classification model is parametrized by only two variables: the threshold $\xi ^{(i)}$ and the mean vector $\pmb {\mu ^{(i)}}$ required for the calculation of distance to the cluster $X^{(i)}$ (defined next in Eqs. 4 and 5). The threshold is not manually defined, but calculated as the maximum distance of all the samples in $X^{(i)}$, increased by a safety margin to the decision boundary $\rho$,

2 $$\begin{aligned} \xi ^{(i)} = (1+\rho ) \cdot \max \limits _{\pmb {x} \in X^{(i)}}\left( d(\pmb {x}, X^{(i)}) \right) . \end{aligned}$$

We used a fixed value of $\rho =0.4$ in our experiments.

Equation (1) provides a decision on the authenticity of a PUF by examining a single image reconstruction. This process is slightly prone to data acquisition-related variability. In order to improve robustness of outcomes, we acquire more than one images for a given engraving and obtain the final decision by majority voting. Thus, if $Y=\left\{ \pmb {y}_1, \pmb {y}_2, \ldots ,\pmb {y}_k \right\}$ is a set of *k* feature vectors obtained from the same engraving, the decision on the engraving’s authenticity is based on $f(Y; X^{(i)}, \xi ^{(i)}) = sign \left( \sum _{\pmb {y} \in Y} f(\pmb {y}; X^{(i)}, \xi ^{(i)}) \right)$. We evaluated the sensitivity of the method to the number of required images and defined it at $k=50$ for the engravings used in this study.

## Implementation

For the experiments of this paper we have fabricated several engravings and extracted their digital signatures in order to evaluate the power of the PUFs (distinction between different engravings), the resiliency to cloning and the robustness of the PUFs to external perturbations, i.e. the reproducibility between scans of the same engraving. Details on the experimental setup and the data collection process are presented in the next sections, and are followed by the description of the evaluation strategy and the criteria used for quantitative assessment.

### Experimental details

A dataset of 10,000 photographs was obtained from 20 engravings, 10 created with beam *Splitter* mask and 10 with beam *Diffuser* mask. All samples of each category (*Splitters* and *Diffusers*) were manufactured by the laser under identical conditions (8ns pulse duration, 17% power, single shot, 2 passes). In the following we describe the procedure and number of data used for one of the two categories, but we note that the same experiments were repeated for both sets produced by beam *Splitter* or beam *Diffuser* holographic masks in order to compare the performance of those two CGH structures as PUFs engraved on silver. For each engraving, 500 images of size $360 \times 640$ pixels were captured by moving the XYZ stages in a pseudo-random way to assure that the dataset includes all possible configurations of positions in which the laser beam can create a complete holographic image reconstruction. Images were registered to the common template space and cropped within a fixed bounding box of size $132 \times 132$, leading to a size $D=17,424$ after vectorization. The reconstructed images were used to learn the *manifold of ‘S-shaped’ CGH-PUFs* as described in section *Signature encoding with t-SNE* and to reduce data dimensionality to $d=10$ using symmetric t-SNE.

For validation of the method, the obtained dataset was divided into two parts. *Part 1* consisted of engravings 1–7 and was used for CGH-PUF generation and authentication based on a data split strategy, while *Part 2* consisted of engravings 8–10 and was used only for fraud detection. Specifically, *Part 1* was further partitioned in two sets. The first set consisted of 450 samples per engraving and was used to form a database of ‘‘authentic’’ PUFs, for each of which a classification model was created. The second set of *Part 1*, i.e. 50 images from each of the engravings 1–7, was used to evaluate the authentication accuracy of the methodology (vector $\pmb {y}$ in Equation 1). This data partitioning setting led to $(50 \cdot 7) \cdot 7$ authentication tests with $50 \cdot 7$ concerning intra-class comparisons (for evaluation of reproducibility) and $(50 \cdot 6) \cdot 7$ concerning inter-class comparisons (for evaluation of malicious attacks).

Images in *Part 2* were not used at all in the creation of the database of ‘‘authentic’’ PUFs, so that the created image manifold (by t-SNE) did not span the space of those engravings. The aim of this experiment was to use the images obtained from those unknown engravings as test samples for assessment of the probability of physical cloning by a malicious manufacturer. In order to be consistent with the experiments performed with *Part 1*, we selected an equal number of random test images (i.e. 50) per engraving. This led to $(50 \cdot 3) \cdot 7$ additional authentication tests concerning inter-class comparisons. In summary, the number of vectorized images used in the dimensionality reduction step by t-SNE was $m=(450 \cdot 7) + 50 \cdot 10= 3650$ and included images from both *Part 1* and *Part 2*.

Upon signature encoding, the classification models were formed to identify authenticity or counterfeit by calculating the thresholds (Eq. (2)) and the cluster centers for each one of the engravings in *Part 1*.

#### Response to perturbation of reconstruction conditions

To investigate the tolerance of our authentication system to experimental faults, we set up an experiment to test reconstruction of images using beam *Diffuser* design in extreme conditions from a set of engravings. During this particular phase, we placed the HeNe laser beam to a position such that only half of the engraving was illuminated. We performed this test for engravings 7–9, and we added engraving 7 in the signature encoding set (*Part 1*), and engravings 8 and 9 in the dataset for fraud detection (*Part 2*). Figure 5 presents the aggregated results from the evaluation of our framework on engraving 7. For all tests that were performed in accordance with the criteria in the following section, BD7 performed worse than the other *Diffuser* CGH-PUFs, but the results remained within acceptable limits.

### Assessment criteria

**Figure 4:**
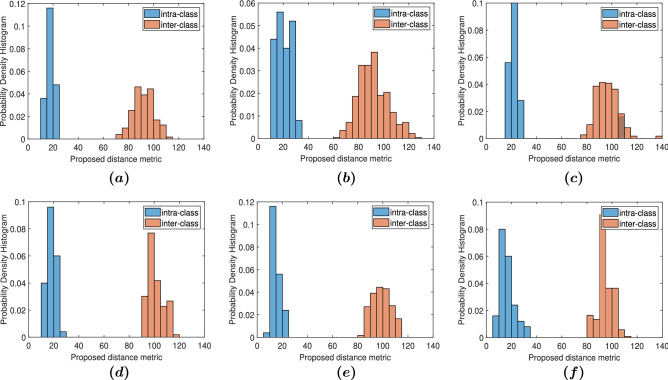
Histograms of inter- and intra-class distance for 3 Engravings of beam *Splitter* (**a**–**c**) and beam *Diffuser* (**d**–**f**) CGH-PUFs, considering the best case (**a**,**d**), the worst case (**c**,**f**) and a randomly selected average case (**b**,**e**) based on their POC estimation.

This section is devoted to the evaluation of CGH-PUFs’ responses regarding their accuracy and their reproducibility. We first computed the intra-class distances within each engraving ($D_{intra}$) and the inter-class distances across engravings ($D_{inter}$) in order to provide a visual assessment of class separability and comparison with work of others. For each engraving the intra- and inter-class distances are calculated in the ${\mathbb {R}}^{d}$ space using the metric of Eq. (5) and visualized as histograms (Fig. 4). Besides histogram plots, we computed 3 quantitative metrics for the CGH-PUFs evaluation: the authentication/classification accuracy, the probability of cloning indicating resiliency in attacks and the robustness assessing reproducibility over time.

- *Authentication accuracy.* The accuracy of PUF authentication was examined in respect to the potential of identifying authentic samples and rejecting counterfeit attempts. The former was assessed by the ratio of original samples that were recognized by our framework as “authentic” (True Positives) and the latter as the ratio of the fake engravings recognized as “authentic” (False Positives). These metrics were used to evaluate the performance of scattering-based PUF-tags on a combination of carrier and taggant materials in a recent optical authentication system^52^.
- *Probability of cloning.* For the estimation of the probability of cloning (POC) of the CGH- PUF by a fraud manufacturer who has knowledge of the conditions of the manufacturing process and the information regarding the holographic mask, we used an approach similar to the methodology presented in^37,53^. Taking into consideration the histograms of inter- and intra-class distances of each engraving, we estimate the POC by fitting a Gaussian distribution on each of the histograms and computing the overlap area. This value corresponds to the amount of fake samples that look more “authentic” than some original samples. POC allows to evaluate the resiliency to attacks without the need to binarize the decision (i.e. use a predefined threshold $\xi$).
- *Robustness.* A complementary metric of equivalent importance for a PUF’s efficiency is robustness. There are various definitions of the robustness in literature^20,21,52,54,55^, depending on the field of application and the category of the PUF, but they can all be summarized as the ability of the PUF to be reproducible and generate the same response when the evaluation process is repeated under the same conditions, without being affected by modifications in the external environment. We therefore measure robustness of the CGH-PUGs by the variation of intra-class distances across repetitions on the authentication process. If the distribution of $D_{intra}$ for specific engraving at time *t* is denoted as $P_{t}$, we quantify any temporal changes through comparison with the intra-class distances distribution of subsequent time points $t+1$ and $t+2$. Two well-known metrics used to measure similarity of probability distributions, the Bhattacharyya distance^56^ and the Kullback–Leibler Divergence^57^.

## Results

### Beam Splitters vs beam Diffusers

To assess the performance of the method, every engraving of *Part 1* obtained by the *Splitter* or *Diffuser* structures was used to create a database of CGH-PUFs and was then validated against the other engravings in *Part 1*. Also, every engraving of *Part 2* was tested against each CGH-PUF in *Part 1*. Table 1a shows the results of all the authentication tests with respect to True Positives and False Positives estimated by our method.

**Table 1:** Results: authentication accuracy and probability of cloning.

(a) Authentication accuracy of *Splitter* and *Diffuser* structures						
*Splitter* CGH-PUFs						
# Engraving	True positives	TP (majority rule)	False positives		FP (majority rule)	
			BS1–BS7	BS8–BS10	BS1–BS7	BS8–BS10
BS1	50/50	1/1	0/300	1/150	0/6	0/3
BS2	50/50	1/1	0/300	0/150	0/6	0/3
BS3	50/50	1/1	0/300	0/150	0/6	0/3
BS4	46/50	1/1	0/300	0/150	0/6	0/3
BS5	50/50	1/1	0/300	0/150	0/6	0/3
BS6	50/50	1/1	0/300	0/150	0/6	0/3

*Diffuser* CGH-PUFs						
# Engraving	True positives	TP (majority rule)	False positives		FP (majority rule)	
			BD1-BD7	BD8-BD10	BD1-BD7	BD8-BD10
BD1	50/50	1/1	0/300	0/150	0/6	0/3
BD2	50/50	1/1	0/300	0/150	0/6	0/3
BD3	50/50	1/1	0/300	0/150	0/6	0/3
BD4	50/50	1/1	0/300	0/150	0/6	0/3
BD5	50/50	1/1	0/300	0/150	0/6	0/3
BD6	50/50	1/1	0/300	0/150	0/6	0/3

(b) Probability of cloning (POC) for BS and BD CGH design						
# Engraving	*Splitter*	# Engraving	*Diffuser*			
BS1	4.4 $\times 10^{-5}$	BD1	8.9 $\times 10^{-14}$			
BS2	3.4 $\times 10^{-7}$	BD2	1.9 $\times 10^{-15}$			
BS3	7.2 $\times 10^{-11}$	BD3	9.6 $\times 10^{-18}$			
BS4	3.0 $\times 10^{-2}$	BD4	2.2 $\times 10^{-17}$			
BS5	2.2 $\times 10^{-5}$	BD5	5.9 $\times 10^{-11}$			
BS6	3.7 $\times 10^{-5}$	BD6	1.5 $\times 10^{-12}$			

Figure 4 illustrates three indicative pairs of histograms selected to visualize good, average and worst classification performance for both CGH structures. The pairs of histograms show the distance of every sample from the cluster center of the engraving it belongs to (intra-class distances) and the distance from the cluster center of every other engraving in the database (inter-class distances), based on the defined metric (methods) respectively. For both CGH structures, there is no overlap between inter- and intra-class distances, with the sole exception of the BS4, where, as it is observed in the Table 1a and in the histogram of Fig. 4c, there is a small group of samples whose the distance metric exceeds the predefined threshold. However, in case of *Diffusers*, the separation of histograms is more evident. The gap between the distributions of inter- and intra-class distances that are presented is equivalent or better comparing to the histogram visualizations that are demonstrated is related work of^21,37,52,55^ based on their similarity indexes.

Following the methodology presented in^37,53^, we used the values of inter- and intra-class distances to estimate the probability of cloning. Table 1b shows the estimated values for *BS* and *BD* design. According to the literature^53^, the acceptance range, that has been used for security applications, is in the order of $10^{-5}$. It is evident that our method achieves significantly lower probability of cloning for all *BD* engravings and lower or comparable POC for *BS* engravings. The *Splitter* structure contains intrinsically a repetitive pattern and, thus, it is less satisfactory for cloning prevention than the *Diffuser*. Observing the deviations of *Splitter* PUFs from the accepted limit, we concluded that engravings with *BD* holographic masks are more suitable for the creation of unclonable security tags and we proceeded next to the evaluation of the *Diffuser* CGH-PUFs.

**Figure 5:**
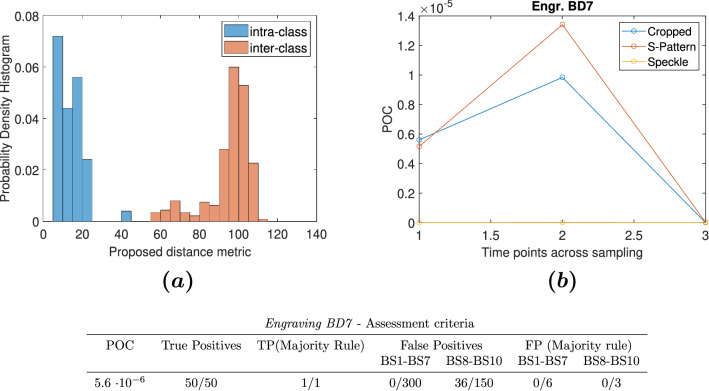
Performance under extreme perturbation of experimental conditions (Engraving BD7): Histogram (**a**) shows intra- and inter-class distances, sub-figure (**b**) shows POC from the 3 time points within a week for cropped images, S-pattern and speckle, and sub-table shows the assessment criteria.

**Figure 6:**
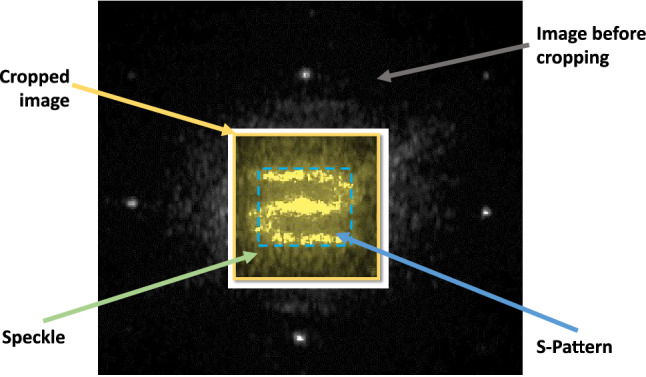
Example illustrating the image segmentation into the S-Pattern area and the pure speckle around it.

**Figure 7:**
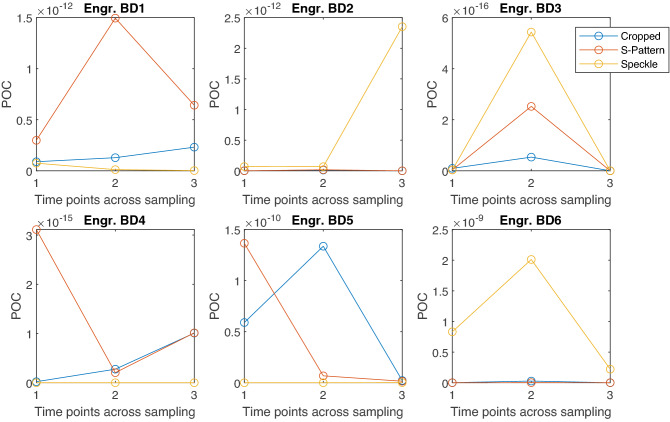
Probability of cloning for the 3 different datasets with cropped images (including S-pattern and speckle), only S-Pattern or only speckle around the pattern, across 3 time points within a week.

### Robustness: S-pattern versus speckle

In addition to the previous tests performed with input the CGH-PUFs obtained after registration and cropping, we also demonstrate some supplementary tests, where every reconstructed image is divided in two parts: the square (central) region of holographic pattern and the speckle noise around it (Fig. 6). The analysis was repeated for every image as well as each of these two subregions separately (Fig. 7). Our aim was to investigate the impact of the introduced holographic pattern to the CGH-PUFs authentication capacity and robustness and to compare our hologram-based approach against techniques that use only speckle pattern.

**Table 2:** Robustness measured by Bhattacharyya distance and Kullback–Leibler (KL) divergence.

# Engraving	Baseline vs 2nd time point			Baseline vs 3rd time point		
	Cropped image	S-Pattern	Speckle	Cropped image	S-pattern	Speckle
**Bhattacharyya distance**						
BD1	1.2 $\times 10^{-2}$	2.6 $\times 10^{-3}$	4.3 $\times 10^{-3}$	1.7 $\times 10^{-2}$	8.1 $\times 10^{-4}$	1.7 $\times 10^{-2}$
BD2	1.6 $\times 10^{-3}$	9.5 $\times 10^{-4}$	9.3 $\times 10^{-3}$	1.8 $\times 10^{-3}$	9.0 $\times 10^{-4}$	4.1 $\times 10^{-3}$
BD3	2.1 $\times 10^{-3}$	9.3 $\times 10^{-4}$	1.2 $\times 10^{-3}$	4.6 $\times 10^{-4}$	1.6 $\times 10^{-2}$	8.0 $\times 10^{-3}$
BD4	7.8 $\times 10^{-3}$	7.4 $\times 10^{-4}$	1.1 $\times 10^{-1}$	3.7 $\times 10^{-3}$	6.2 $\times 10^{-3}$	8.6$\times 10^{-2}$
BD5	5.1 $\times 10^{-4}$	2.0 $\times 10^{-3}$	1.5 $\times 10^{-3}$	6.6 $\times 10^{-3}$	8.1 $\times 10^{-3}$	2.6 $\times 10^{-3}$
BD6	6.2 $\times 10^{-5}$	3.7 $\times 10^{-4}$	2.4 $\times 10^{-3}$	1.9 $\times 10^{-3}$	3.7 $\times 10^{-6}$	6.4 $\times 10^{-4}$
**KL divergence**						
BD1	1.4 $\times 10^{-1}$	6.0 $\times 10^{-2}$	7.4 $\times 10^{-2}$	2.1	7.2 $\times 10^{-2}$	1.9
BD2	2.8 $\times 10^{-2}$	8.6 $\times 10^{-2}$	2.6 $\times 10^{-1}$	5.3 $\times 10^{-2}$	6.0 $\times 10^{-2}$	1.2 $\times 10^{-1}$
BD3	5.0 $\times 10^{-2}$	6.1 $\times 10^{-1}$	1.4 $\times 10^{-1}$	2.9 $\times 10^{-2}$	4.2	1.5 $\times 10^{-1}$
BD4	8.5 $\times 10^{-2}$	5.1 $\times 10^{-2}$	2.5	5.0 $\times 10^{-2}$	2.3 $\times 10^{-1}$	2.57
BD5	1.3 $\times 10^{-2}$	2.3 $\times 10^{-2}$	8.4 $\times 10^{-2}$	3.5 $\times 10^{-2}$	5.2 $\times 10^{-1}$	2.4 $\times 10^{-1}$
BD6	1.9 $\times 10^{-2}$	2.1 $\times 10^{-1}$	3.0 $\times 10^{-2}$	2.7 $\times 10^{-2}$	5.9 $\times 10^{-3}$	4.2 $\times 10^{-1}$

To quantify the robustness, we performed the experiments in the same readout regime, by capturing 50 images per engraving for 2 more days within a week and compare their intra-class distances with the results of the initial experiment. Table 2 demonstrates the robustness assessed through the Bhattacharyya distance and KL Divergence based on the histogram of $D_{intra}$ per engraving. A value of zero (0) for both Bhattacharyya distance and KL divergence indicates identical distributions. The robustness level estimated by both metrics reveals high similarity between the data of the baseline measurements and those from the additional experiments.

## Discussion

The presented PUF generation and authentication methodology is based on sample-to-cluster distances evaluated in a low dimensional space. In one-class classification problems most techniques use only the ‘‘authentic’’ class dataset and identify deviations as intrusions. Since our approach relies on signature encoding with t-SNE, it is more efficient if a large number of samples (from different engravings) are used for manifold learning, in order to better express relations and pairwise similarities between the different subspaces and optimize the decision boundary (threshold $\xi$).

The outcomes of analysis assessing authentication accuracy, probability of cloning and robustness demonstrate the high potential of CGHs as PUFs. This computational image processing and representation approach allows to quantify the differences among various engravings. A comparison between beam *Splitter* and beam *Diffuser* holographic structures reveals that the use of the latter increases the tolerance of the CGH-PUFs to counterfeit attacks, possibly due to the characteristic periodic pattern of a *BS* structure which makes image reconstruction less sensitive to any minor alterations of the material after the laser micromachining process. Consequently, the captured reconstructions tend to be more similar among the engravings, a fact that is imprinted on the results, where *BS* CGHs appear to have higher probability of cloning, especially the engraving BS4 (Table 1b).

The results of the *BD* CGH-PUFs show remarkable performance during the analysis. All the engravings demonstrate a probability of cloning which is much lower than the recommended upper limit for security applications. Concerning the analysis of the intentionally perturbed experiment of the engraving BD7, we have tested the sensitivity of the method to strong perturbations of the data acquisition procedure. Although the acquired images were not complete, as only a partial view of the image reconstruction was obtained on the readout setup, the method succeedes in distinguishing the authentic versus fake responses. In the presented results (Fig. 5), BD7 shows 36 False Positives, which belong to BD8 and BD9 engravings, whose reconstructed images were also partially obtained. Nevertheless, despite the acquisition-related artifacts, our method presents good performance, as all the POC estimations for the BD engravings (Table 5b) are above the acceptable limit.

In optical PUFs literature, a typical approach for cryptographic-authentication applications utilizes speckle noise as distinctive factor for modelling and encoding of the PUFs^37^. It has been observed that random speckle pattern, generated from laser illumination over heterogeneous, semi-transparent materials, is a secure optical PUF. The problem that arises in case of the experiments that capture exclusively speckle noise is the sensitivity of the setup to changes of the external environment (e.g. changes in background lightning conditions add indistinguishable noise). Our framework utilizes CGH-PUFs that contain in addition to speckle noise a predefined holographic patter. This distinctive pattern is useful in guiding the image registration algorithm in the preprocessing step (since it allows to find spatial correspondences) and therefore helps to correct any misplacement errors during image acquisition.

Moreover, this holographic pattern is encoded in the low-dimensional data signature along with the speckle noise information. To determine if there is any benefit of its presence, we investigated the POC and robustness of our system within a time span of a week on two different image sub-regions: the square enclosing the holographic pattern and the speckle noise area around it (Fig. 6). The obtained POC values (Fig. 7) show that in half of the cases (engravings) the S-pattern sub-region provides lower POC, compared with the total image. The superiority of the S-Pattern is observed in robustness Table 2, where, compared to the speckle sub-region, it shows to be a little more stable during the experiments across time points. The Bhattacharyya Distance values indicate that there are more similarities across time points for the S-Pattern sub-region than for the speckle sub-region, or both parts together (cropped image) for all the engravings, except BD5. KL Divergence appears to have similar performance for cropped image and S-pattern, but in the case of speckle, it seems less stable, especially from baseline experiments to the 3rd time point.

With respect to potential replication threats it should be noted that although the laser engraved CGH structures for a single item in a production line could be potentially replicated on soft substrates, such as Polydimethylsiloxane (PDMS), this could only be replicated using a multistep nanoimprint lithography on non-metallic bulk substrates like e.g. polymers. Therefore the proposed method of fabrication of intrinsic optical PUFs is suitable for hard materials, like silver, rather than soft materials.

The ease in the fabrication of extrinsic PUFs is reflected on the overall fabrication cost. However as mentioned before, extrinsic PUFs suffer from vulnerability to attacks. The cost benefits of laser fabrication of intrinsic optical PUFs presented here, can be better appreciated when compared to existing intrinsic PUF solutions found in the literature, namely electronic PUFs and silicon PUFs. In both cases, these are fabricated using standard photolithographic methods that are multistep, more expensive and complex than laser methods. On the other hand, the proposed method is an one step method that uses a low cost nanosecond industrial laser with minimal (nearly zero) running and maintenance costs. The PUF requirement for randomness in the fabrication process minimizes the initial acquisition cost since the laser source should be unstable both in terms of intensity and polarization and the galvo system should introduce uncertainty in the positioning of the laser beam.

Another advantage of this work is the simple and easy to incorporate readout setup. Compared to other more complicated and time-consuming procedures^20,26^, our authentication system is not based on expensive laboratory equipment, neither requires high expertise or increased workload and it can be implemented in a compact setup by a non-expert user.

## Conclusion

We presented a novel, simple, low-cost and industrial friendly system for strong intrinsic PUF in the form of CGH-PUFs. The proposed methodology of laser fabricated PUFs combined with sophisticated image analysis techniques delivers an authentication mechanism for strong anti-counterfeiting. CGH-PUFs are essentially a speckle-based technique that, however, overcome limitations of present day similar approaches that suffer from low robustness due to sensitivity of speckle on experimental conditions in the read out. The CGH-PUFs exhibit low probability of cloning and a good level of robustness while retaining the merit of simplicity both in fabrication and read out. We anticipate that our method will impact the proliferation of PUFs in everyday commodity items.

## Methods

### Design of CGH

The CGH is designed as a binary phase only hologram using the commercially available software VirtualLab Fusion by LightTrans^41^. The CGH is designed to work as beam *Diffuser* or beam *Splitter*. In particular, the CGH design procedure is based on an optimization process using the classical Gerchberg–Saxton algorithm^58^. The overall goal of this process is to find the optimal phase mask for a target image (in our case the ‘S’ pattern) by minimizing a cost function (in our case the Mean Square Error) for a given set of parameters, such as wavelength of the light source, fabrication feature size, phase levels, to name a few. The output of this optimization process is the phase mask that is exported as a bitmap image and subsequently engraved on the silver plate.

### Experimental setup

We fabricated intrinsic optical PUFs in the form of holograms on silver using a ns infrared (1070 nm) fiber laser system developed by Sisma SpA, with galvo mirrors scanner and a f-theta lens (f = 100 mm). Silver is a high reflectivity precious metal, that it is very hard to engrave. The first demonstration of laser engraving of holograms on silver was presented in^59^. Here, we follow the fabrication protocol outlined in^59^. For the image reconstruction, the sample was positioned in a XYZ linear stage (Aerotech ANT130). The holographic image was captured by a low-cost web camera, with an adjusted red filter in front of its lens. The distance between the laser output and the sample is 38 cm while the distance between the sample and the reconstruction plane is 74 cm. The whole process is automated using Aerotech motion Composer and Matlab Software. Our experiment was performed in a light proof box with minimal background light and illuminated with a typical HeNe Laser emitting at 633 nm.

### Image standardization

To transform image to grayscale, we used the luminosity method^60^, which weighs R, G and B components according to their wavelengths, to define the coefficients for the calculation of the gray-scale image intensity values *I*:

3 $$\begin{aligned} I = 0.299 \cdot R + 0.587 \cdot G + 0.114 \cdot B \end{aligned}$$

### Signature encoding with t-SNE

During manifold learning with t-SNE, the default value ($p=30$) was used for the perplexity of the Gaussian kernel which indicates the effective number of local neighbors of each point and is employed in the computation of joint probabilities in t-SNE. We also note that, as the original dimensionality *D* was very large, we first performed *Principal Component Analysis* (PCA)^61^ reducing the dimensionality to 50 before applying t-SNE. Since this step is intended to only remove linear correlations among variables, the selection of a precise number of principal components is not critical, as long as the selected value is large enough to retain the majority of original variance in the data.

### Authentication rule: distance metric

To encode the variation of a CGH-PUF representation, $X^{(i)}$, that includes the different image manifestations of a single engraving *i* scanned on various time points, we use the cluster center $\pmb {\mu }^{(i)} \in {\mathbb {R}}^{d}$ calculated as sample mean:

4 $$\begin{aligned} \pmb {\mu ^{(i)}} = \frac{1}{n}\sum _{j=1}^{n}\pmb {x}^{(i)}_j \end{aligned}$$

We define the probability of a new test sample $\pmb {y}$ to have been acquired from engraving *i* to be inverse proportional to its Euclidean distance from the cluster center $\pmb {\mu }^{(i)}$, which is given by the following equation:

5 $$\begin{aligned} d(\pmb {y}, X^{(i)}) = \sqrt{\sum _{k=1}^{d}(y_{k}-{\mu }_{k}^{(i)})^2}. \end{aligned}$$

### Robustness

**(a) Bhattacharyya distance** is widely used in various research domains (feature extraction and selection, image processing, etc.) for measuring class separability^62^. Assuming that the intra-class histograms follow Gaussian distribution with mean $\mu$ and variance $\sigma ^{2}$, the Bhattacharyya distance $D_{B}$ between $P_{t}$ (reference point) and $P_{t+1}$ (next time point), is calculated as

6 $$\begin{aligned} D_{B}(P_{t},P_{t+1})= \frac{1}{4}ln\Bigg (\frac{1}{4}\bigg (\frac{\sigma _{P_{t}}^{2}}{\sigma _{P_{t+1}}^{2}} +\frac{\sigma _{P_{t+1}}^{2}}{\sigma _{P_{t}}^{2}}+2\bigg )\Bigg )+\frac{1}{4}\Bigg (\frac{(\mu _{P_{t}}-\mu _{P_{t+1}})^{2}}{\sigma _{P_{t}}^{2}-\sigma _{P_{t+1}}^{2}}\Bigg ). \end{aligned}$$

The experiments were repeated two times within a week ($t = \{1, 2\}$).

**(b) Kullback–Leibler (KL) Divergence** also known as relative entropy, is a measure of how one probability distribution is different from another (reference) probability distribution^57,63,64^. It is used to calculate how much information is lost when we approximate one distribution with another and represents the expectation of the logarithmic difference between the two probabilities:

7 $$\begin{aligned} D_{KL}(P_{t}||P_{t+1}) = \sum _{i\epsilon {\mathcal {X}}}^{}P_{t} \cdot log\bigg (\frac{P_{t}}{P_{t+1}}\bigg ) \end{aligned}$$
